# Editorial: The Plant Holobiont Volume II: Impacts of the Rhizosphere on Plant Health

**DOI:** 10.3389/fpls.2021.809291

**Published:** 2021-12-20

**Authors:** Nadia Lombardi, Sheridan Lois Woo, Francesco Vinale, David Turrà, Roberta Marra

**Affiliations:** ^1^Department of Agricultural Sciences, University of Naples Federico II, Naples, Italy; ^2^Center for Studies on Bioinspired Agro-Environmental Technology (BAT Center), Naples, Italy; ^3^Department of Pharmacy, University of Naples Federico II, Naples, Italy; ^4^Task Force on Microbiome Studies, University of Naples Federico II, Naples, Italy; ^5^Institute for Sustainable Plant Protection, National Research Council, Naples, Italy; ^6^Department of Veterinary Medicine and Animal Productions, University of Naples Federico II, Naples, Italy

**Keywords:** microbiome, microbial biodiversity, endophytes, insects, induced resistance, plant metabolites, plant pathogens

The concept of “holobiont” introduced by Margulis (1991), as a simple biological unit involving a host and a single inherited symbiont, today assumes a more general meaning recognizing that microorganisms are universally present on and associated with multicellular eukaryotic organisms. The plant “holobiont” represents an assembly of the host with its symbiotic microorganisms in interaction, that may occur internally or be associated with the host tissue, acting as a biological hub able to replicate and transmit genetic information (Zilber-Rosenberg and Rosenberg, 2008). Thus, the soil community represents a reservoir of various microbes from which the plant can selectively retrieve a specific microbiome to meet its requirements. Numerous microorganisms are able to perform biological and physiological functions for the plant, such as nutrient cycling and water distribution, that significantly impact soil properties, plant development and health. Different microorganism groups represent key components in the soil-plant systems directly involved in the network of interactions occurring in the rhizosphere, inner plant tissues and phyllosphere (Barberán et al., 2012; Hassani et al., 2018). Soil and plant microbiomes have an important role in plant development and in soil health, providing a secondary genome that codes for the genetic machinery activated in biosynthetic processes regulating ecological and biological functions. Moreover, beneficial microbes can influence plant health and productivity by improving tolerance to stress through modulation of different functional traits, thus affecting quality and safety of plant products (Timmusk et al., 2017; Compant et al., 2019).

The composition of each microbiome is influenced by a myriad of factors, including environmental, soil physical properties, availability of nutrients, and associated plant species. Among the research papers included in this Research Topic, Seitz et al. studied the influence of climate changes connected with permafrost thaw on the composition of the soil microbiome. Significant differences were found in the taxonomy of the microbial species associated with the different active layers of soil in the permafrost thaw gradient. It was revealed that microbes associated with highly disturbed soils, negatively affected productivity of several boreal plant species. Di Lelio et al. demonstrated that varying temperatures (20 and 25°C) affected diversely the development of two *Trichoderma* species applied to tomato plants that subsequently resulted in a different induced defense response of the plants to the aphid *Macrosiphum euphorbiae* and the noctuid moth *Spodoptera littoralis*. Although the mechanisms activated in the plant by the microbiome are not yet fully understood, there is substantial evidence that many microbes provide beneficial effects to crops including disease control, improved nutrient acquisition, and stress tolerance. Zheng et al. revealed that the key species in suppressive soil and/or healthy tobacco plants were fundamental in the inhibition of soil-borne pathogens, such as *Ralstonia solanacearum*. The authors found that healthy plants have more complex bacterial networks than diseased plants, and that many potential beneficial bacteria, having inhibitory effects on the pathogen, included isolates related to Proteobacteria and Actinobacteria, were found in the disease-suppressive soil. Moreover, the high abundance of *Fusarium* in the conducive soil and infected roots was correlated to the occurrence of bacterial wilt disease.

Soil microorganisms establish close relationships with the host plant during mycorrhizal symbiosis. The review by Li et al. presented an updated perspective on the importance of the interaction between orchids and orchid mycorrhizal fungi (OMF). OMF involvement in key functions such as development, distribution and dynamics, environmental adaptability and protection against different soil pathogens was reported. The authors suggested that changes in the structure and quantity of OMF may have important consequences on orchid distribution and health.

The composition of the root microbiome changes according to the root compartment association. The work of Illescas et al. analyzed the effect of inorganic N (calcium nitrate-CAN) top dressing applications, *T. harzianum* T34 and their combination on wheat root microbial community composition. It was highlighted that there was an overall differentiated bacterial and fungal composition in bulk soil, rhizosphere and root endosphere. In all three compartments, the bacterial diversity was always higher than that recorded for fungi, and the microbial biodiversity decreased from bulk soil to root endosphere. Moreover, the combination of CAN and T34 affected to a greater extent the bulk soil bacterial levels in comparison to the individual applications, while the fungal composition was notably affected by each treatments assayed.

Anzalone et al. obtained 400 bacterial isolates from different tomato root-associated compartments (the soil close to the root surface, the root surface, and internal the root), and tested their ability to solubilise phosphates, produce siderophores, and act as antagonists. The authors highlighted that the sampling site influenced bacterial composition more than the root partition.

The development of the holobiont concept is now providing a new scientific basis for variation genetics, which is heritable and offered by the plant microbiome, in particular by the endophytic compartment (Nogales et al., 2016). The review by Harman et al. analyzed the beneficial effects of endophytes on plants, providing examples indicating improved nutritional status, photosynthetic capability and maintenance of an efficient internal cellular functioning. Moreover, it was explained that the mechanisms involved in the activation of plant resistance to diseases and abiotic stresses occur through the production of Symbiont-Associated Molecular Patterns, microbial elicitors that interact with receptors in plant cell membranes, thus resulting in signal transduction *via* MAP kinase and modification in plant gene expression and physiology.

Varga et al. utilized x-ray fluorescence spectromicroscopy and proteomics to demonstrate that two diazotrophic endophytes promoted organic and inorganic phosphorus uptake in poplar roots. Carro-Huerga et al. used scanning electron microscopy and confocal scanning laser microscopy analyses, to verify the presence of *Trichoderma* TI154, 6 weeks after inoculation, in parenchyma fibers and xylem vessels of grapevine wood, confirming the endophytic colonization by the beneficial fungus and its ability to limit the development of the pathogen *Phaeoacremonium aleophilum*, the causal agent of Grapevine Trunk Diseases.

The specific interactions that occur between plant species and the soil microbial community are also important due to the differential influence on plant growth and resistance to stress. There is a reciprocal exchange whereby the plants influence the microbiome composition in the surrounding soil, and in turn the microbial complex modulates the growth and protection of the plants. This interaction may involve different plant species that can recruit the microbes that can suppress plant pathogens, and consequently stimulate plant growth. In this context, there is a growing interest in applying and transplanting soil microbiomes in order to enhance plant performance during cultivation. Howard et al. tested four different crop species that were inoculated with diverse soil microbiomes, originating from actively-managed agricultural fields or native unplanted fields, and analyzed their growth and resistance to two insect pests. Soil microbiomes derived from late succession plant communities increased maize, cucumber, tomato, and lettuce resistance to *Trichoplusia ni*, in contrast to those crops treated with microbiomes from agricultural sources, although the same application failed to provide protection of tomato against *Spodoptera frugiperda*. In addition, plant growth promotion effects were found to be species-specific and depended upon the different microbiomes used. This evidence was also confirmed by Choi et al. who demonstrated that the upland soil microbial fraction increased the resistance to *R. solanacearum* in tomato cultivar Hawaii 7996, but not in the susceptible cultivar Moneymaker. In addition, the resistance of Hawaii 7996 to bacterial wilt was lost when the transplanted microbiota was heat-killed.

The MiSeq sequencing performed by Wang et al. provided new insights on transplantation as feasible approach for controlling soil-borne diseases in *Panax ginseng* plants over long growth periods. The authors found that the abundances of some pathogenic bacteria and fungi, as well as the functional richness associated with nutrient elements, decreased with increasing age of transplanted ginseng plants.

Plants can influence their microbiomes by producing and releasing various metabolites in their surrounding environment. In addition, when subjected to a stress condition the generated compounds can regulate the structure of the plant microbiome. The diverse manners that plant secondary metabolites may shape plant microbiome have been reviewed by Pang et al. They focused on the recent developments in analytical methodologies and multi-omics, providing an excellent overview of the networks between the co-operating partners which have implications on sustainable crop productions.

In their study, Wei et al. demonstrated that the total fungal biomass was significantly higher in the rhizosphere of *Verticillium* infected cotton plants, as compared to the rhizosphere of healthy plants, represented by an increase in different saprophytes and a decrease in mycorrhizal fungi. The diseased plants showed a ratio of total fungi to total bacteria much higher than in the healthy plants, suggesting a modification of the root microbial community due to the stress condition.

All the contributions collected in this Research Topic strengthen the already known relevance of microbiomes in the management of a sustainable agriculture. We hope this Research Topic of articles will encourage new researches on this subject of current increasing interest and importance.

## Author Contributions

NL wrote the first draft of the manuscript. RM, FV, DT, and SW revised and improved the manuscript. All authors defined the subject of this Research Topic, joined in the editing procedure, and approved this Editorial for publication.

## Funding

This work was supported by MISE CRESO (Grant number Protection n. F/050421/01-03/X32), PRIN 2017 (Grant number PROSPECT 2017JLN833), MISE Sportello Agrifood DM 5/3/2018 (Grant VIABio), European Union Horizon 2020 Research and Innovation Program, ECOSTACK (grant agreement no. 773554), MUR, PNR 2015-2020. ARS01_00985 BIOFEEDSTOCK – Sviluppo di Piattaforme Tecnologiche Integrate per la Valorizzazione di Biomasse Residuali PSR Veneto 16.1.1 (Grant number Divine n. 3589659), PSR Campania 2014/2020 Misura 16 - Tipologia di intervento 16.1 Azione 2 Sostegno ai Progetti Operativi di Innovazione Progetto DI.O.N.IS.O., C.U.P. B98H19005010009.

## Conflict of Interest

The authors declare that the research was conducted in the absence of any commercial or financial relationships that could be construed as a potential conflict of interest.

## Publisher's Note

All claims expressed in this article are solely those of the authors and do not necessarily represent those of their affiliated organizations, or those of the publisher, the editors and the reviewers. Any product that may be evaluated in this article, or claim that may be made by its manufacturer, is not guaranteed or endorsed by the publisher.
